# Current‐Induced Domain Wall Dynamics in Pt/Co/Ni/Co Nanoribbons With Curvilinear Cross‐Sections

**DOI:** 10.1002/adma.74919

**Published:** 2026-09-24

**Authors:** Tiange Dong, Jitul Deka, Chi Fang, André M. A. Farinha, Yicheng Guan, See‐Hun Yang, Stuart S. P. Parkin

**Affiliations:** ^1^ Max Planck Institute of Microstructure Physics Halle Germany

**Keywords:** 3D racetrack memories, chiral domain walls, current‐induced domain wall motion, curvilinear magnetism, multiphoton lithography

## Abstract

Recent advances in compact and readily fabricated magnetic thin film devices have created great interest for their application in magnetic memory technologies, in particular, racetrack memories. However, most work to date has been carried out using two‐dimensional racetracks but the full potential of racetrack memory can only be achieved with three‐dimensional architectures. A range of 3D geometries can significantly influence magnetic interactions through spatial curvature. In this work, we investigate nanoribbons with curvilinear surfaces onto which Pt/Co/Ni/Co multilayers are formed by sputter deposition techniques and the consequent current‐induced domain wall dynamics. Nanoribbons with controllable cross‐sectional curvatures are formed by advanced multiphoton lithography. We show that the surface curvature, whether concave or convex, can either enhance or suppress the current‐induced domain wall motion. These different responses arise from Oersted fields generated by the current flowing through these curvilinear geometries. In these structures, up‐down and down‐up domain walls move at the same velocity under current injection. However, by introducing more complex hybrid structures that incorporate both concave and convex sections across the nanoribbon's width, we show that up‐down and down‐up domain walls have distinct velocities, and consequently can act as a domain wall filter.

## Graphical abstract

3D curvilinear magnetic racetrack nanoribbons are formed with concave and convex cross‐sections using an advanced multi‐photon 3D printer. The curvature is shown to strongly affect current‐induced domain wall motion along the nanoribbons. In nanoribbons with cross‐sections that have both concave and convex segments, the velocities of domain walls with different configurations (up‐down and down‐up) are distinct giving rise to a domain wall filter.

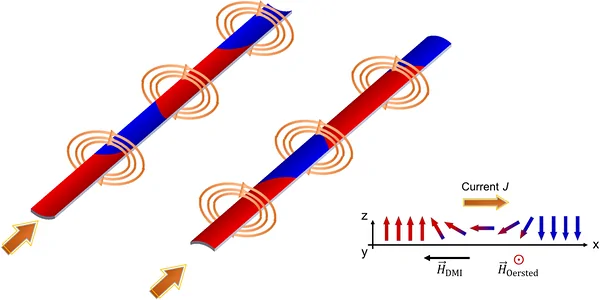

## 1 Introduction

The current‐induced high‐speed domain wall (DW) motion in magnetic multilayered thin films that form “racetrack” nanowires has attracted considerable interest in recent years. Of particular interest are heavy metal/ferromagnetic (HM/FM) multilayers with perpendicular magnetic anisotropy (PMA) that have been shown to display chiral Néel DWs through an HM/FM interface‐derived Dzyaloshinskii–Moriya interaction (DMI) [1, 2, 3] that arises from broken inversion symmetry. The Néel DWs have the same chirality for both DW configurations (up‐down ⨀⨂ and down‐up ⨂⨀) [4, 5]. In such systems, spin–orbit torques (SOTs), arising from spin current injection due to a spin Hall effect (SHE) in the HM layer, have been shown to drive DWs in magnetic nanowires at very high velocities [1, 6, 7, 8]. Nearly all these studies to date have been carried out with horizontal racetracks, that is, 2D structures.

Three‐dimensional geometries provide an additional route to tailor magnetic interactions and, consequently, the current induced motion of DWs. Experiments have shown that when magnetic thin films are formed on curved or non‐planar surfaces, the curvilinear geometry can modify the magnetic energy by introducing effective anisotropic and chiral exchange interactions [9, 10, 11, 12, 13, 14, 15, 16]. Theoretical studies show that such energy modifications can strongly influence the equilibrium DW configurations [17, 18, 19, 20], DW pinning [21, 22], and current‐driven DW dynamics [23, 24, 25, 26]. In perpendicularly magnetized nanowires hosting Néel‐type DWs, geometrical curvature or torsion can lift the energy degeneracy between ⨀⨂ and ⨂⨀ DWs, acting as an effective exchange field and giving rise to non‐reciprocal DW transport [27, 28, 29]. In addition, current‐induced Oersted fields associated with cylindrical or curved nanowire cross‐sections have been proposed as another mechanism for manipulating DW motion [30, 31, 32]. Despite that, there have been few experimental studies of current‐induced DW motion (CIDWM) in curvilinear nanowires.

In this work, we prepare magnetic nanoribbons having various curved cross‐sections to systematically investigate the influence of such curvature on CIDWM. We utilize an advanced multiphoton lithographic (MPL) technique for device fabrication [27]. Compared to other commonly used methods, such as self‐assembly [33, 34], flexible bending [35, 36], and free‐standing membrane transfer [37], MPL allows more precise control over the detailed geometry. We demonstrate that the curvilinear geometrical modifications affect the CIDWM.

## 2 Results

### 2.1 Fabrication of the 3D Curvilinear Nanoribbons

Curvilinear nanoribbons having concave and convex cross‐sections are fabricated using advanced MPL. The fabrication process builds 3D structures by slicing the target structures into a series of planar layers and then sequentially building the structure one layer at a time. Each of these layers is fabricated by moving a highly focused laser beam through a liquid polymeric mixture with an optically active component. A femtosecond pulsed laser (780 nm, 80 MHz repetition rate) is used. The laser beam results in polymerization of the liquid to form a solid. In this manner, complex 3D structures can be fabricated, as shown schematically in Figure 1a. A glass substrate, through which the laser beam is passed from the rear‐side, is used as the printing substrate, and the individual layers are set to be ∼10 nm thick. This spacing is smaller than the voxel size (∼50 nm) [27], thereby allowing for structures with high‐precision cross‐sectional profiles (Figure S2).

**FIGURE 1:**
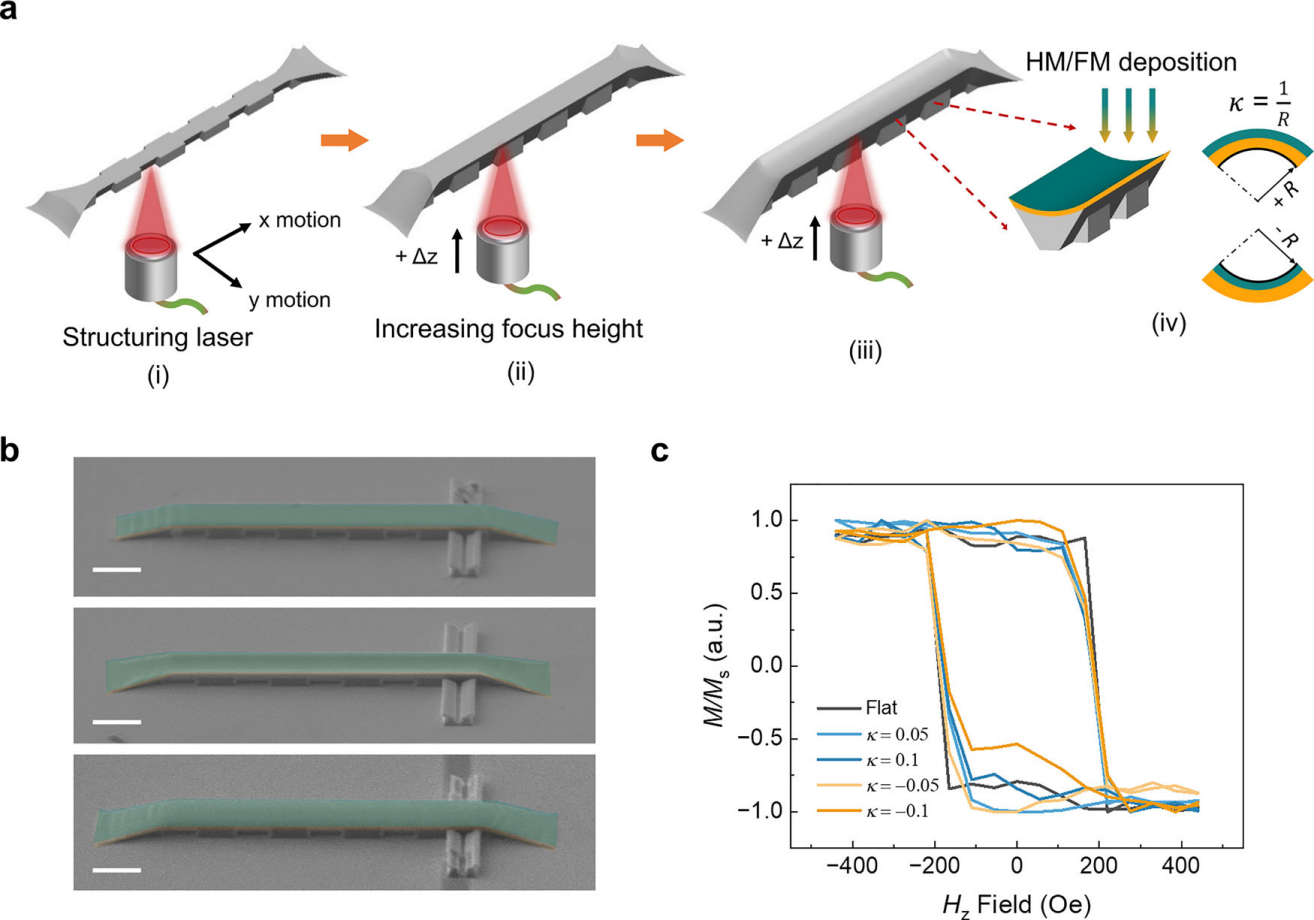
Nanoribbon fabrication and characterization. (a) (i)–(iii) Layer‐by‐layer printing of the 3D structures using MPL. After printing one layer, the focal point is raised by Δ*z* = 10 nm for the next layer. (iv) Deposition of the HM/FM thin film by sputtering: the HM layer (yellow) and FM layers (green). The concave or convex surface of the nanoribbon provides for cross‐sectional curvature. The nanoribbon is V‐shaped to avoid deposition on the sidewalls. κ corresponds to the curvature of the nanoribbon. (b) Scanning electron microscope (SEM) characterization of a (top) flat, (middle) concave, and (bottom) convex cross‐sectional curvilinear ribbon from a 52° sample tilt viewpoint, where the lateral sections on the right sides are isolation bars. The racetrack nanoribbons are colored in yellow for the HM layer and green for the FM layers, and are connected to the substrate by the left and right ramps. Uncolored images are shown in Figure S1. Note that the substrate is also covered by the deposited HM/FM thin films, but is divided into distinct electrical contact pads through the usage of isolation bars and subsequent FIB milling. SEM scale bar: 5 µm. (c) MOKE hysteresis loops extracted from the nanoribbon surface, where different colors correspond to samples with different curvatures. κ has units of µm^−1^. All samples exhibit a similar coercivity *H*_C_.

The nanoribbons are designed with a V‐shaped profile as shown in Figure 1a (iv). This design helps to prevent coating of the nanoribbons’ sidewalls during the deposition of the magnetic layers. The magnetic thin film is formed from ultra‐thin layers of Pt/Co/Ni/Co that are deposited by magnetron sputtering. This stack gives rise to significant PMA as well as chiral Néel‐type DWs [2, 3]. At one end of the 45 µm long nanoribbon, 6.5 µm‐long straight isolation bars with V‐shaped profiles (Figure 1b) are fabricated. These bars define the contact pads for electrical access. Focused ion beam (FIB) milling is then used to extend the isolation bars and separate the pad areas, ensuring that the 3D nanoribbon can be electrically accessed without the need for any additional connections to the magnetic layers deposited onto the nanoribbon itself (see Figure S1). The curvature κ of the nanoribbon cross‐section is varied from one device to another, thereby, creating flat (κ = 0), convex (κ = +0.1 µm^−^^1^) and concave (κ = −0.1 µm^−^^1^) nanoribbons (see Figure 1a(iv),b). The curvature is defined as $\kappa =\frac{1}{R}$, where *R* is the radius of the curved surface of the nanoribbon. The lateral width of the nanoribbon was fixed at 4 µm, which was chosen to provide sufficiently large changes in CIDWM whilst allowing for robust optical detection of the DWs [38, 39]. The lateral dimensions can be further reduced to well below sub‐micron dimensions using our state‐of‐the‐art MPL system (see Figure S10). Magnetic hysteresis loops are measured for each device under an out‐of‐plane (OOP) external magnetic field using a magneto‐optical Kerr effect (MOKE) microscope. Figure 1c shows five magnetic hysteresis loops which are similar for flat, convex and concave nanoribbons. All loops show good squareness with no obvious difference in coercivity (*H*_C_), indicating that the magnetic properties on the polymeric scaffold surface are similar in each case. Note that the OOP coercivity of the film deposited directly on the glass substrate is much smaller (∼50%), presumably due to the smoother surface (see Figure S9).

### 2.2 Current‐Induced Domain Wall Motion

CIDWM in nanoribbons with flat, convex, and concave curvatures are measured using MOKE microscopy (Figure 2a). The electrical current flows from left to right so that a positive DW velocity corresponds to motion from left to right. Prior to the CIDWM measurements, DWs are nucleated and positioned at the left or right end of the curvilinear nanoribbons by applying one of the following procedures. Method (i): (a) a large OOP magnetic field is applied to magnetize the entire structure in one direction, (b) an OOP magnetic field, just below the coercivity of the magnetic nanoribbon is then applied in the opposite direction, thereby nucleating a DW within the contact pad. The nucleated DW moves along the ramp driven by the applied field till it reaches the nanoribbon itself. Method (ii): applying an in‐plane (IP) field directed along the nanoribbon and current pulses simultaneously that generates DWs at the ramps. Due to the tilted magnetization at the ramps with respect to the sample normal direction and heating by current pulses, the ramps act as efficient DW nucleation sites. Importantly, since the IP field is aligned along the nanoribbon direction, only one DW configuration, either ⨀⨂ or ⨂⨀, is selectively introduced depending on the IP field orientation (see Figures S11 and S12). Once a DW is nucleated and injected into the curvilinear ribbon section, the external fields are switched off unless otherwise needed for measurements (e.g., DMI measurements).

**FIGURE 2:**
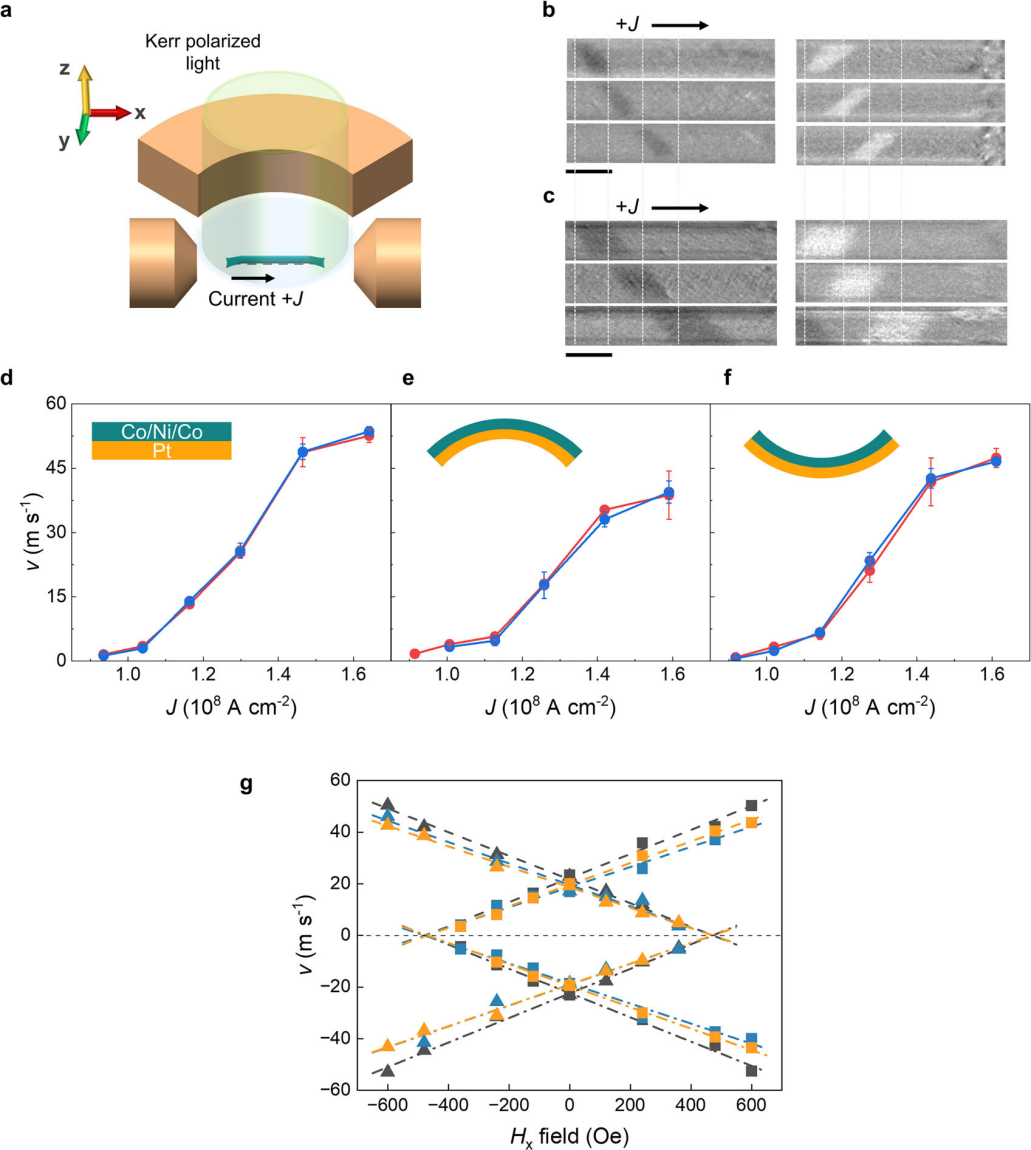
Current‐induced DW motion. (a) Schematics of the measurement setup of the MOKE microscope with the ring (OOP) magnet and head‐to‐head (IP) magnet, both of which are highlighted in gold. MOKE images of the DW motion are taken from (b) convex (κ = +0.1 µm^−1^) and (c) concave (κ = −0.1 µm^−1^) nanoribbons with *J *= 1.42 × 10^8^ A cm^−^^2^ and a sequence of eight current pulses. The black (left) and white (right) contrasts indicate the displacement of ⨀⨂ and ⨂⨀ DWs, respectively. The dashed lines in (b) and (c) indicate the DW displacement after each series of current pulses in the convex device as a reference. Scale bars: 5 µm. (d–f) DW velocity *v* at different drive currents *J* for (d) flat, (e) convex, κ = +0.1 µm^−1^, and (f) concave κ = −0.1 µm^−1^ nanoribbons. Data for ⨀⨂ DWs are shown in red and those for ⨂⨀ DWs are shown in blue. On the concave ribbons, the DW motion was measured to be faster than on the convex ribbons. (g) DMI measurement from the dependence of the DW velocity on the x‐directional IP field *H*_x_ on flat (black lines), convex (blue lines), and concave (yellow lines) nanoribbons, and for ⨀⨂ DWs (triangular dots) and ⨂⨀ DWs (rectangular dots). The dashed and dash‐dotted lines represent +*J* and −*J*, respectively. The DMI fields were measured at *J* = ±1.27 × 10^8^ A cm^−^^2^. Note that the same set of nanoribbons was used in (b) to (g).

Figure 2d–f shows the CIDWM velocities for ⨀⨂ and ⨂⨀ DWs on flat, convex‐, and concave‐curvature nanoribbons, respectively. Interestingly, we find that the DW velocity *v* depends on the curvature κ. For the concave nanoribbons (Figure 2f), the DWs exhibit slightly faster CIDWM for both DW configurations (⨀⨂ and ⨂⨀) than the convex (Figure 2e) counterparts at +*J*. The same effect is found for the opposite current polarity (−*J*) (Figure S13). Note that the DW itself becomes tilted across the nanoribbon under the influence of current. The DW tilts in opposite directions for ⨀⨂ and ⨂⨀ DW configurations [40, 41]. We find that the magnitude of the DW tilt angle, χ, is affected by the sign and magnitude of the curvature, where positive or negative χ correspond to counterclockwise or clockwise tilting of the DW. Figure 2b,c display Kerr images of the CIDWM for ⨀⨂ and ⨂⨀ DWs for convex and concave nanoribbons, with the same curvature magnitude, respectively. At a current density, *J* = 1.42 × 10^8^ A cm^−^^2^, the average χ measured from the convex nanoribbons is χ∼+37° ±  2° (− 38° ±  1°) for ⨀⨂ (⨂⨀) DWs, whereas for the concave nanoribbons χ ∼ +30° ± 1° (− 29° ±  1°) for ⨀⨂ (⨂⨀) DWs, which are smaller than those of the convex counterparts. Note that χ ∼ +32° ±  2° (− 32° ±  3°) for ⨀⨂ (⨂⨀) DWs on flat ribbons (see Figure S3). We find that the DWs remain tilted even after the current pulses are switched off [7, 28, 42], as previously observed for 2D racetracks. This is ascribed to DW pinning.

The DMI that determines the chiral Néel‐like character of the DW is reflected in an effective DMI field, ${\vec{H}}_{\text{DMI}}$, that is perpendicular to the DW and takes opposite signs for ⨀⨂ and ⨂⨀ DWs [2]. Such ${\vec{H}}_{\text{DMI}}$ can be measured by applying a longitudinal IP magnetic field *H*_x_ along the nanoribbon axis (x‐direction) and measuring the CIDWM as a function of the strength and direction of this *H*_x_ field. The strength and direction of the DMI field corresponds to the value of *H*_x_ where the DWs do not move. Note that the curvilinear samples show a slight decrease in the DW velocity, *v*, and a smaller slope in the *v* versus *H*_x_ curves shown in Figure 2g, as compared to the flat sample. The intercepts of the extrapolated *v* versus *H*_x_ curves indicate DMI fields of 474 ±  1 Oe for the flat ribbon, 475 ±  3 Oe for the convex ribbon (κ = +0.1 µm^−1^), and 472 ±  5 Oe for the concave ribbon (κ = −0.1 µm^−1^). Since the measured DMI values are nearly constant irrespective of the curvature, we find that the interfacial DMI is unlikely to be responsible for the curvature‐induced velocity modulations. We will show that other curvature‐induced effects play a key role in our observations, as discussed below.

### 2.3 Structures of Hybrid Curvilinear Cross‐Sections

To further investigate the role of 3D cross‐sectional curvature, hybrid nanoribbons in which two halves with curvatures of opposite signs are fabricated, as shown in Figures 3a and S1. The 3D structure fabrication and thin film deposition procedures are identical to single‐curvature ribbons. Two types of devices are investigated: convex curvature on the left and concave curvature on the right (denoted CV/CC) and vice versa, concave curvature on the left and convex curvature on the right (CC/CV) (see Figure 3a). Only one cross‐sectional curvature of such nanoribbons was evaluated, namely, κ = +0.1 µm^−1^ (CV half) and κ = −0.1 µm^−1^ (CC half), the same curvature as that used in the single curvature nanoribbons, discussed in Figure 2.

**FIGURE 3:**
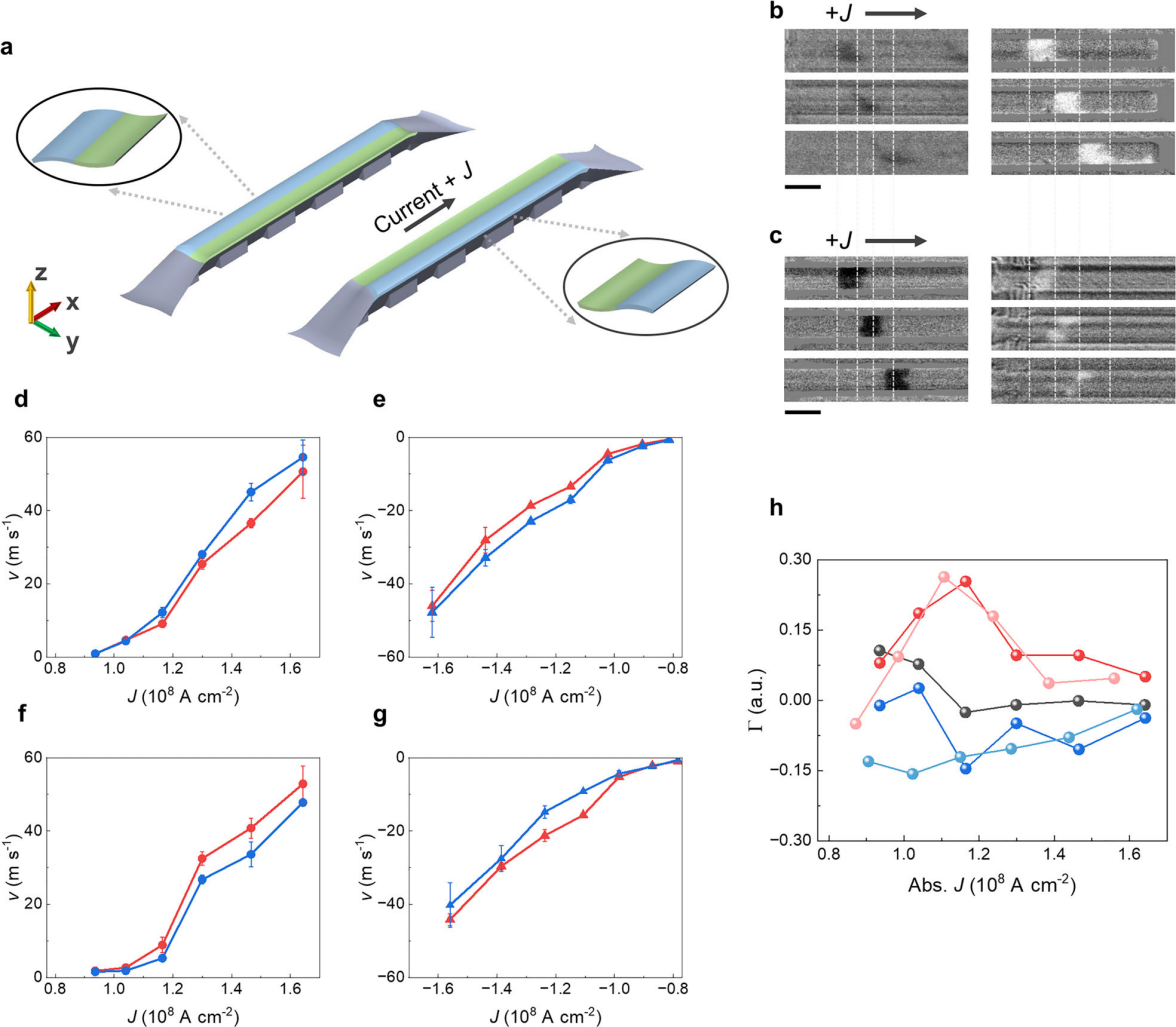
DW motion in hybrid nanoribbons. (a) Schematics of CV/CC (left) and CC/CV (right) hybrid nanoribbons and the direction of +*J*. The color illustrates segments of opposite κ in the 3D structure: CV segment (blue) and CC segment (green). The insets illustrate cross‐sectional views of the nanoribbons. The measured DW motion and tilting are shown in (b) for the CV/CC and (c) for the CC/CV nanoribbon, respectively, where differences are observed between ⨀⨂ (black) and ⨂⨀ (white) DWs for their displacement and tilting. The images are taken at *J *= 1.46 × 10^8^ A cm^−^^2^ with a sequence of eight pulses between each frame. Scale bar: 5 µm. DW velocity *v* versus *J* is measured for (d) +*J* and (e) −*J* for the CV/CC nanoribbons, respectively, and (f) +*J* and (g) −*J* for the CC/CV nanoribbons, respectively. Different colors are used for ⨀⨂ (red line) and ⨂⨀ (blue line) DWs. Different symbols are used for +*J* (circular dots) and −*J* (triangular dots). Here, ⨀⨂ and ⨂⨀ DWs show enhancement or suppression of their velocity corresponding to less or more tilting of the DW. (h) Normalized velocity difference Γ between ⨀⨂ and ⨂⨀ DWs. The red and blue lines show Γ for CC/CV and CV/CC devices, respectively. The two lighter‐colored curves indicate Γ under −*J* polarity. The black curve indicates Γ for the flat device as a reference.

Interestingly, as distinct from the single curvature nanoribbons discussed above, nanoribbons with hybrid curvatures show distinct DW velocities for ⨀⨂ and ⨂⨀ DWs. For the CV/CC ribbons, for both +*J* and *−J*, the ⨂⨀ DWs move faster than the ⨀⨂ DWs (Figure 3d,e), whereas the ⨀⨂ DWs move faster than the ⨂⨀ DWs for the CC/CV ribbon (Figure 3f,g). This is consistent with symmetry whereby we expect that −*J* applied to ⨀⨂ (⨂⨀) DWs on a CV/CC nanoribbon should correspond to +*J* applied to ⨂⨀ (⨀⨂) DWs on a CC/CV nanoribbon, and vice‐versa.

To quantitatively evaluate the curvature induced changes in DW velocity, we define a normalized velocity difference, $\Gamma =\frac{v_{⊙⊗}-v_{⊗⊙}}{v_{⊙⊗}+v_{⊗⊙}}$. As shown in Figure 3h, *+J* and −*J* measurements show comparable Γ values for both CC/CV and CV/CC devices. A maximum Γ is observed near *J* ∼ 1.15 × 10^8^ A cm^−^^2^ with *v* ∼ 10 m s^−1^. At low *J*, variations in Γ are likely due to local pinning effects [27, 43]. At higher *J* (i.e., *J* > 1.3 × 10^8^ A cm^−^^2^), Γ saturates at approximately ± 0.05–0.1 for the CC/CV and CV/CC devices, corresponding to a relative maximum velocity contrast of about 10%–20% between the ⨀⨂ and ⨂⨀ DWs.

Another interesting point is that χ is reduced to nearly zero for ⨂⨀ (⨀⨂) DWs on a CV/CC (CC/CV) nanoribbon, as compared to the significant tilting discussed above for both flat and single‐curvature ribbons (Figures 2 and S3). For the opposite DW configurations: ⨀⨂ (⨂⨀) DWs on a CV/CC (CC/CV) surface, the tilting angle is rather increased to +44 ± 2° (− 42 ± 4°). We note that the current‐driven DW velocity is strongly affected by the DW tilt, increasing for reduced tilt angles and decreasing for larger tilt angles, as discussed further below.

### 2.4 Geometrical Effect on Nanoribbons With Convex and Concave Cross‐Sections

Let us discuss the underlying mechanism of the distinct DW velocities for nanoribbons with convex and concave cross‐sections. For the devices studied here, the very similar values of *H*_c_ (Figure 1c) measured for the flat, convex, and concave devices suggest that the strain and strain gradient in the magnetic layers are too small to substantially modify the magnetic anisotropy [34, 36, 44] or chirality [45, 46] (see Supporting Information Note 2 for details). Whilst curvature‐induced changes to exchange and dipole interactions can, in principle, modify the DW energy and magnetization [12, 13], such modifications become more pronounced for large κ [13, 26]. Thus, these contributions are likely small for the devices considered here (Supporting Information Note 3). Rather, we find that curvature‐induced modifications of the Oersted fields arising from the current flowing along the nanoribbons can account for our observations: the Oersted fields along the y‐direction, $H_{\mathrm{Oe}}^{y}$, are modified by the cross‐sectional curvatures, thereby acting differently for DWs at the top surface of nanoribbons with convex and concave curvatures of the same magnitude (Supporting Information Note 3 and Figures S19–S21).

To investigate this further, the Oersted field distributions in flat, convex‐, and concave‐curvature nanoribbons are calculated using the geometrical parameters of our devices for various current densities, see Figure S5. Cross‐sectional maps of $\;H_{\mathrm{Oe}}^{y}$ for flat, convex, and concave nanoribbons are presented in Figure 4b,c for a current density of *J *= 1.5 × 10^8^ A cm^−^^2^. Note that the current‐induced Oersted fields are pointing to the right (+$\hat{y}$) at the top of the nanoribbon and pointing to the left (−$\hat{y}$) at the bottom of the nanoribbon for +*J* (see Figure 4a). We focus on the Oersted fields at the top surface of the nanoribbon, as shown in Figure 4e, where the DW motion occurs. For a flat ribbon, the Oersted field at the top surface is uniform with a field strength of 58 Oe at the middle. For the convex ribbon, the Oersted field is enhanced and reaches ∼63 Oe at the middle and drops near the edges. In contrast, for the concave ribbon, the Oersted field at the middle is decreased to ∼53 Oe and increased near the edges. Thus, DWs that move on a convex or concave ribbon experience a difference in the Oersted field.

**FIGURE 4:**
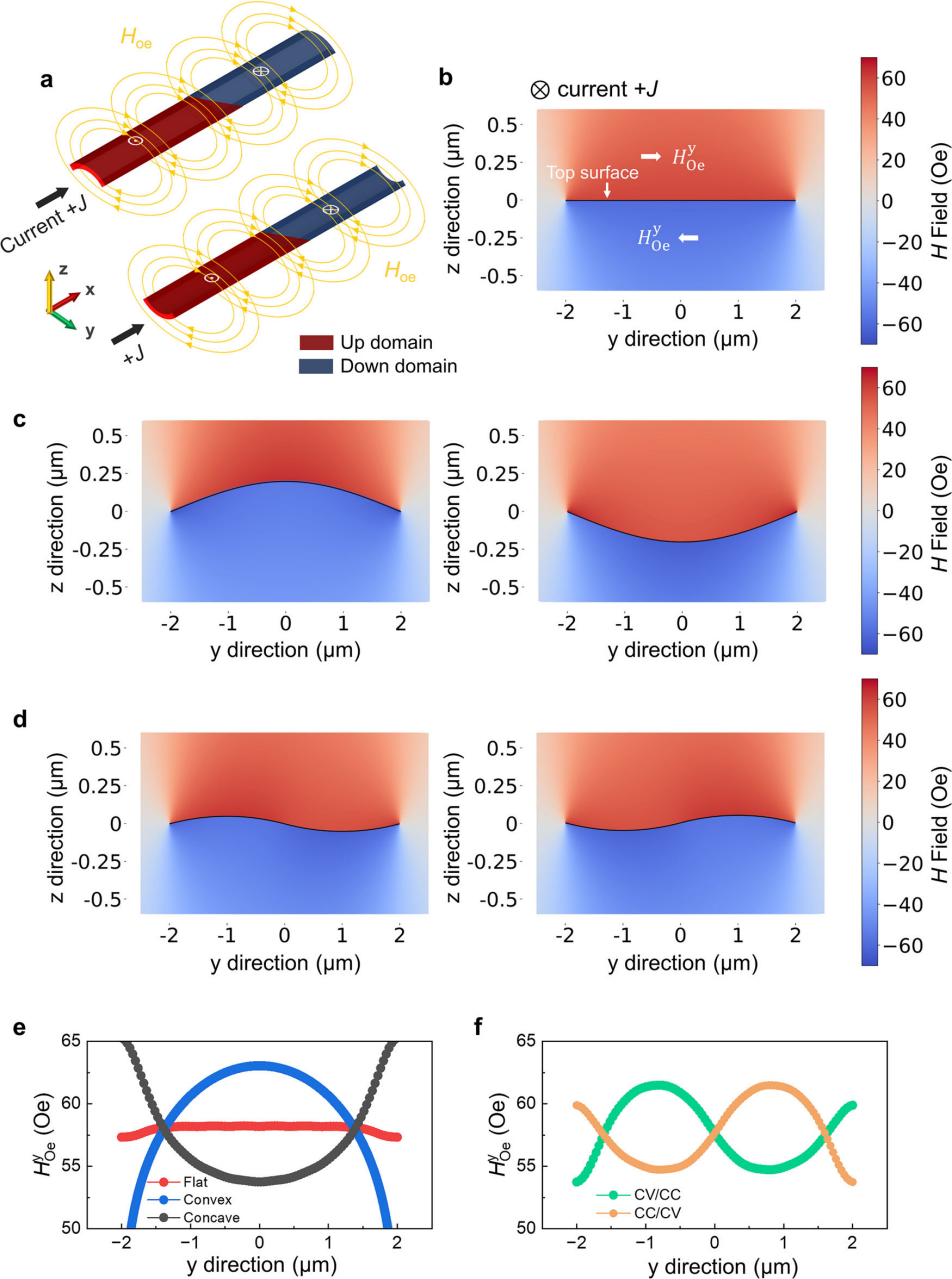
Calculations of the y‐directional Oersted fields. (a) Schematics of the current‐induced Oersted field *H*_Oe_ around a convex‐ (top) and a concave‐curvature (bottom) racetrack nanoribbon. The y‐directional Oersted fields $H_{\mathrm{Oe}}^{y}$ are calculated for (b) flat, (c) convex (left) and concave (right), and (d) CV/CC (left) and CC/CV (right) nanoribbons, where the black line shows the cross‐sectional geometry of the corresponding nanoribbon. $H_{\mathrm{Oe}}^{y}$ is modified by the cross‐sectional geometry depending on the magnitude and sign of the curvature. Calculations of $H_{\mathrm{Oe}}^{z}$ are shown in Figure S6. (e,f) Show $H_{\mathrm{Oe}}^{y}$ at the nanoribbon top surfaces. For all panels (b–f), positive $H_{\mathrm{Oe}}^{y}$ corresponds to the field direction pointing to the right, and negative $H_{\mathrm{Oe}}^{y}$ corresponds to the field pointing to the left. A reversed curvature exhibits a different $H_{\mathrm{Oe}}^{y}$ at the top surface.

Calculated maps of $H_{\mathrm{Oe}}^{y}$ for hybrid ribbons (CV/CC and CC/CV) are shown in Figure 4d. As for the single curvature nanoribbons, $H_{\mathrm{Oe}}^{y}$ at the top surface is enhanced (∼62 Oe) within the convex‐curvature segment and decreased (∼54 Oe) within the concave‐curvature segment, as shown in Figure 4f. Reversed curvatures show reversed Oersted field profiles. Subsequently, DWs that move on these hybrid ribbons are influenced by non‐monotonic lateral Oersted fields, either enhanced or decreased depending on the convex or concave geometries for the same curvature magnitude.

### 2.5 Interplay of the Geometrical Effect and DW Dynamics

Let us discuss how cross‐sectional curvatures affect CIDWM. Our calculations discussed above show that convex and concave curvature cross‐sectional geometries give rise to distinct profiles of $H_{\mathrm{Oe}}^{y}$. To understand the CIDWM, we incorporate $H_{\mathrm{Oe}}^{y}$ into the Landau–Lifshitz–Gilbert (LLG) equation that is given by:

(1) $$\frac{\partial \vec{m}}{\partial t}=-\gamma \vec{m}\times {\vec{H}}_{\text{eff}}+\frac{\alpha }{m}\vec{m}\times \frac{\partial \vec{m}}{\partial t}+\frac{\gamma H_{\mathrm{SHE}}}{2}\vec{m}\times \vec{m}\times \hat{y}\;$$

where $\vec{m}$ is the magnetization, α is the Gilbert damping, γ is the gyromagnetic ratio, *H*_SHE_ is the effective spin Hall parameter acting along $\hat{y}$, and the effective field​ ${\vec{H}}_{\text{eff}}$ contains contributions from ${\vec{H}}_{\text{DMI}}$, the magnetic anisotropy field ${\vec{H}}_{k}$, and the current‐induced Oersted field ${\vec{H}}_{\text{Oe}}$:

(2) $${\vec{H}}_{\text{eff}}={\vec{H}}_{\text{DMI}}+{\vec{H}}_{k}+{\vec{H}}_{\text{Oe}}$$

*H*_SHE_ is proportional to the current density *J* and the spin Hall angle θ_SH_ (see Supporting Information Note 1). Note that ${\vec{H}}_{\text{Oe}}$ corresponds to $H_{\mathrm{Oe}}^{y}$. Contributions from spin‐transfer torques (STT) are not considered here since the spin Hall torque (SHE‐SOT) is typically much larger for thin films when the spin Hall angle and DMI are large [27].

Thus, three dominant torques determine the DW velocity in our system: the spin‐orbit torque ${\vec{\tau }}_{\text{SOT}}$, the DMI torque ${\vec{\tau }}_{\text{DMI}}$, and the Oersted torque ${\vec{\tau }}_{\text{Oe}}$:

(3) $${\vec{\tau }}_{\text{SOT}}=\frac{\gamma H_{\mathrm{SHE}}}{2}\vec{m}\times \vec{m}\times \hat{y}\;$$

(4) $${\vec{\tau }}_{\text{DMI}}=-\gamma \vec{m}\times {\vec{H}}_{\text{DMI}}$$

(5) $${\vec{\tau }}_{\text{Oe}}=-\gamma \vec{m}\times {\vec{H}}_{\mathrm{Oe}}^{y}$$

${\vec{\tau }}_{\text{SOT}}$ acts so as to rotate $\vec{m}$ along $\hat{y}$. ${\vec{H}}_{\text{DMI}}$, that is perpendicular to the DW and IP, is a unidirectional field that is induced by the Pt/FM interface, thereby giving rise to left‐handed Néel‐type walls. Note that the orientations of ${\vec{H}}_{\text{DMI}}$ are opposite for ⨀⨂ and ⨂⨀ configurations since the DMI is a chiral field [2, 5]. When current pulses move the DWs, the sign of the DW tilting angle χ is opposite for ⨀⨂ and ⨂⨀ DWs [29, 40, 42]. The magnitude of χ is largely determined by the competition between the DMI and dipole energies [7, 41, 47] (Supporting Information Note 3).

Figure 5a–h illustrates how the varying Oersted field and χ influence the torques for convex and concave nanoribbons. For flat nanoribbons, the DMI and Oersted torques are identical for ⨀⨂ and ⨂⨀ DWs. However, the combination of the field‐like torques (${\vec{\tau }}_{\text{FL}}={\vec{\tau }}_{\text{DMI}}+{\vec{\tau }}_{\text{Oe}}$) are increased (decreased) in concave (convex) nanoribbons (Figure 5c,d), thereby moving the DWs faster in concave as compared to convex nanoribbons (Figure 5a,b), for the same values of κ and *J*. Our analytical model of DW motion with ${\vec{H}}_{\mathrm{Oe}}^{y}$ that, as discussed earlier, is distinct for concave, flat, and convex nanoribbons, as shown in Figure 6. The parameters in the model correspond to the device geometries and the measured properties of the thin films (see Supporting Information Note 1 and Table S1). In Figure 6a,b, a large ${\vec{\tau }}_{\text{Oe}}$ reduces *v* (= $\dot{q}$, where *q* is the DW position) for convex devices, since it acts in opposition to ${\vec{\tau }}_{\text{DMI}}$, whereas a small ${\vec{\tau }}_{\text{Oe}}$ increases *v* for concave devices. Note that ${\vec{\tau }}_{\text{DMI}}$ remains the dominant contribution to ${\vec{\tau }}_{\text{FL}}$ and is identical for the convex and concave geometries, while the contribution of ${\vec{\tau }}_{\text{Oe}}$ depends on the sign of the curvature. A larger ${\vec{H}}_{\mathrm{Oe}}^{y}$ increases the magnitude of χ while a smaller ${\vec{H}}_{\mathrm{Oe}}^{y}$ decreases it (see Figure 6c,d). Note that ${\vec{\tau }}_{\text{FL}}$ for ⨀⨂ and ⨂⨀ DWs is the same for the same curvature and, therefore, has the same effect on *v* and χ. Note that the calculated differences in *v* and χ between concave and convex ribbons match our experimental trends [27, 28].

**FIGURE 5:**
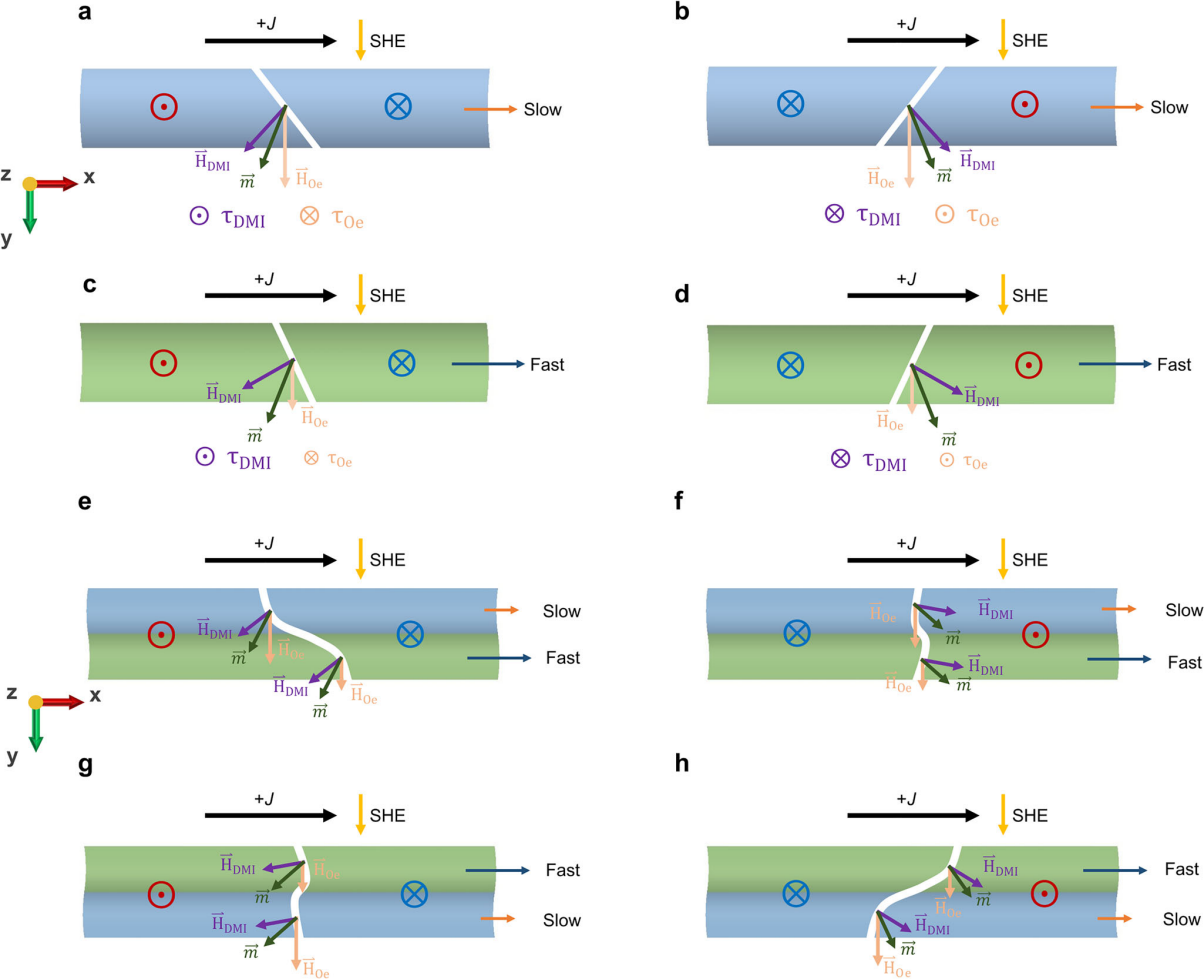
Illustration of the curvature effect on the current induced DW velocity and tilting. The magnetization vector $\vec{m}$, the Oersted field ${\vec{H}}_{\text{Oe}}$, the DMI field ${\vec{H}}_{\text{DMI}}$, and the spin Hall injection are plotted with colored arrows. The same colors are used for the corresponding torques. The DW is represented as the white line between the domains with a tilt angle χ. (a–d) show the influence of the convex (blue) and concave (green) cross‐sectional geometry on the ⨀⨂ or ⨂⨀ DWs. For a single‐curvature nanoribbon with fixed κ, ${\vec{H}}_{\text{Oe}}$ acts equivalently on ⨀⨂ and ⨂⨀ DWs. As a result, the two DW configurations exhibit nearly identical velocities for the same CC or CV geometry. The velocity difference between CC and CV nanoribbons originates from their distinct curvature‐modified ${\vec{H}}_{\text{Oe}}$, which leads to different ${\vec{\tau }}_{\text{Oe}}$. (e–h) illustrate the cases for the CV/CC and CC/CV nanoribbons. These geometries have a different influence on the ⨀⨂ and ⨂⨀ DWs, depending on whether the CV or CC segment is on the left or on the right. Their effect is attributed to the combination of the Oersted field‐induced velocity difference on different segments and the intrinsic tilting of the DW, which modifies the tilting overall and results in a complex shape of the DW. For negative current polarity, see Figure S7.

**FIGURE 6:**
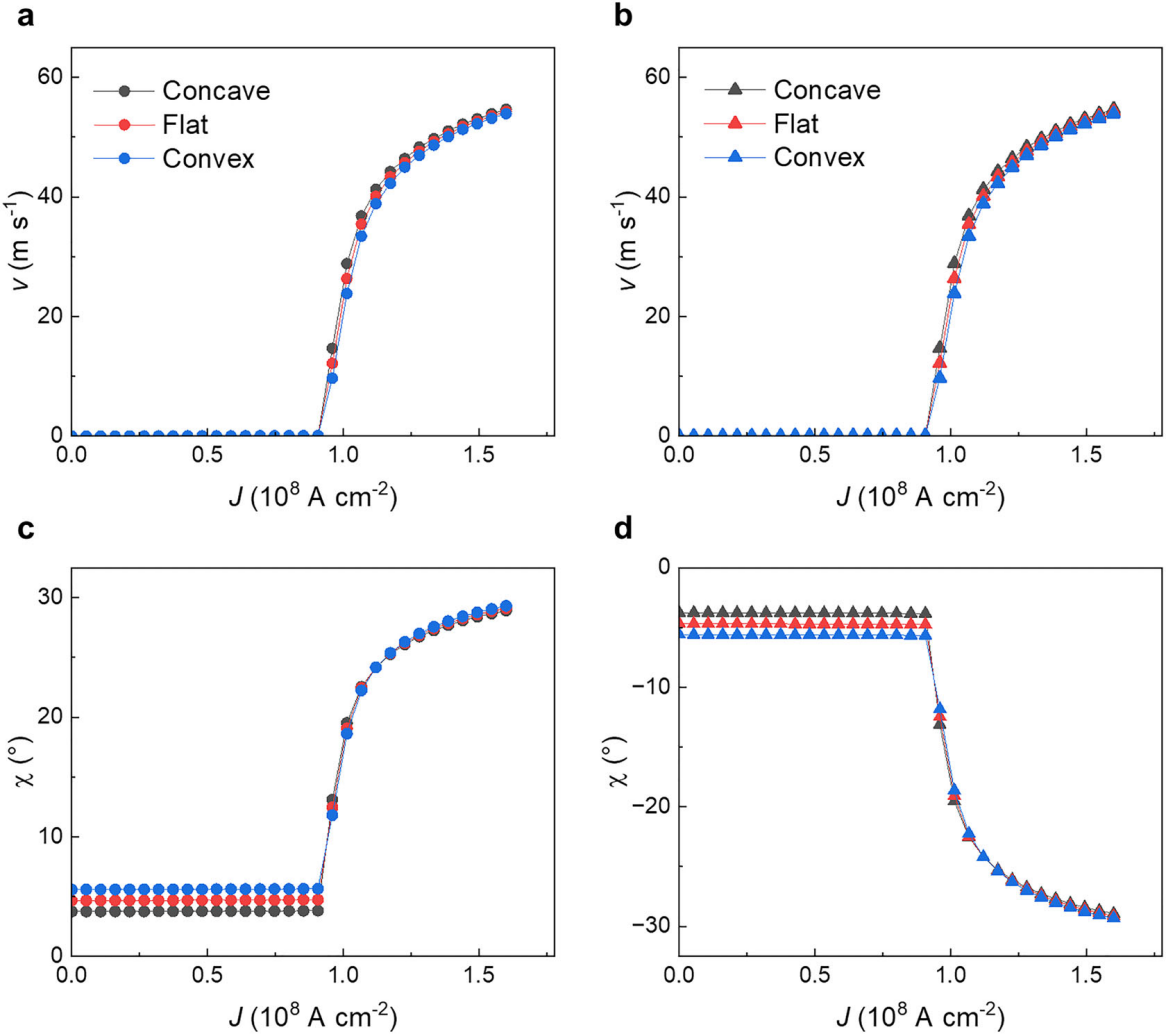
Calculations of the DW velocity and tilting. (a,c) show the velocities *v* and tilting angle χ for the ⨀⨂ DWs (circular dots), and (b,d) show the velocities and tilting for the ⨂⨀ DWs (triangular dots), on the flat, concave (κ = −0.1 µm^−1^), and convex (κ = +0.1 µm^−1^) nanoribbons. Both ⨀⨂ and ⨂⨀ DWs show a gradual increase in velocity when the cross‐sectional geometry is changed from convex to flat to concave. Meanwhile, a slight reduction is shown in the tilting angle of the DWs for *J* > 1.25 × 10^8^ A cm^−2^ when changing the curvature. Note that the tilting angles of the ⨀⨂ and ⨂⨀ DWs have opposite signs corresponding to counter‐clockwise and clockwise tilting of the DW. The results for *−J* are shown in Figure S8.

Next, we discuss hybrid structures of the form CV/CC and CC/CV (Figure 5e–h). The non‐monotonic variation of ${\vec{H}}_{\mathrm{Oe}}^{y}$ across the CC and CV segments is illustrated in Figure 4f, as discussed previously. First we discuss the case for ⨀⨂ DWs and +*J* (Figure 5e,g). Here, the intrinsic counterclockwise tilting of the DWs, combined with the velocity contrast between the faster‐moving CC segment and the slower‐moving CV segment, gives rise to a geometry‐controlled mechanism for the overall DW motion. When the CC segment is positioned on the left and the CV segment on the right (CC/CV structure), the inter‐segment drag compensates the intrinsic DW tilting, leading to a reduced overall tilt. In contrast, reversing the segment order (CV/CC structure) enhances the DW tilt. For the ⨂⨀ DWs, the direction and magnitude of the tilt is opposite (Figure 5f,h). Thus, the tilt of ⨂⨀ DWs is reduced in the CV/CC structures and enhanced in the CC/CV structures. In both cases, negative current has an identical effect (Figure S7). The DW velocity is faster for the smaller tilt and slower for the larger tilt. This can be seen in our DW model that shows the highest DW velocities arise when $\vec{m}$ is orthogonal to ${\vec{H}}_{\text{DMI}}$ because this maximizes ${\vec{\tau }}_{\text{DMI}}$ and minimizes ${\vec{\tau }}_{\text{Oe}}$, which is optimized for small χ, whereas increasing χ decreases the torque and, thus, reduces the DW velocity. This leads to another interesting characteristic for the hybrid nanoribbons, which are distinct from the single‐curvature nanoribbons: ⨀⨂ and ⨂⨀ DWs travel at the same velocity in the latter case but travel at different velocities in the hybrid nanoribbons due to the interplay of the magnetic chirality of the DW and the geometrical chirality of the segments.

## 3 Conclusion

In summary, we have investigated the influence of cross‐sectional curvature on the current‐induced DW dynamics in perpendicularly magnetized Pt/Co/Ni/Co multilayer nanoribbons. By utilizing advanced MPL to fabricate 3D structures with precisely defined cross‐sectional curvatures, we analyze how the curvature effects modulate the CIDWM. Our results show that micron‐sized nanoribbons with a concave curvature promote faster DW motion than their counterparts with a convex curvature for both ⨀⨂ and ⨂⨀ DWs irrespective of the current direction. This difference originates from lateral Oersted fields generated by the current that are increased or decreased depending on the curvature, whether concave or convex. Even more interestingly, hybrid structures formed with both CC and CV segments lead to differences in the DW velocities whether the CC segment is on the left or on the right of the nanoribbon. The differing local Oersted fields enhance or suppress the tilting of the ⨀⨂ or ⨂⨀ DWs, thereby decreasing or increasing the DW velocity. Our findings offer direct evidence that 3D cross‐sectional curvatures can be engineered to control chiral DW motion, and suggest new strategies for creating velocity filters that discriminate between ⨀⨂ and ⨂⨀ DWs.

## 4 Methods

### 4.1 3D Structure Fabrication

Three‐dimensional structures were fabricated using a femtosecond Er fiber laser system (Menlo Systems C‐Fiber 780) operating at a wavelength of 780 nm, with a repetition rate of 80 MHz. The fiber laser was integrated into a modified stimulated emission depletion microscopy setup (Abberior Instruments). The laser light was coupled from the bottom side and intially focused on the top surface of the borosilicate glass substrate for printing, see Figure S2. An objective lens with a 100× magnification and a 1.45 numerical aperture was used. The laser beam was scanned at high speed in the x‐y plane using a galvo‐mirror. The between‐layer height was controlled by a xyz piezostage (P‐545, Physik Instrumente). The photoresist was formed from 0.25 wt% 7‐diethylamino‐3‐thenoyl‐coumarin (DETC, Luxottica Exciton) dissolved in pentaerythritol tetraacrylate (PETTA, Sigma‐Aldrich). The pulse energy for the fabrication process was 0.18 nJ and the laser scan speed was fixed at 6.25 mm s^−1^. After laser exposure, the 3D structures were developed in propylene glycol monomethyl ether acetate (PEGMEA, Sigma‐Aldrich) for 5 min, followed by rinsing in isopropanol for 5 min to remove any residual developer.

### 4.2 Magnetron Sputtering

After the 3D structures were formed, they were coated with HM/FM multilayers formed from Pt/Co/Ni/Co (50/3/7/3, layers in Å). A physical vapor deposition system MANGO (multi‐source, atomically engineered, next generation, alloys and compounds deposition system) was used, which has a base pressure of <10^−9^ Torr. Magnetron sputter deposition was carried out at ambient temperature using Ar at a pressure of 3 mTorr. A 120 Å thick TaN layer was first deposited as an underlayer for the Pt/Co/Ni/Co film. A TaN capping layer of 50 Å was deposited to protect the Pt/Co/Ni/Co film from oxidation. Growth rates of 0.9 Å s^−1^ for Pt and 0.2 Å s^−1^ for the Co and Ni layers were used.

### 4.3 Electrical Isolation and Sample Characterization

After depositing the magnetic films on the 3D structures, electrical contact pads were formed using FIB milling. A Tescan GAIA3 FIB with an in‐situ SEM was used. SEM imaging was performed at an electron beam energy of 5 keV, while milling was carried out with a 30 keV Ga^+^ ion beam. The FIB milling was carried out starting from the ends of the V‐shaped isolation bars. The ends are positioned away from the nanoribbon to prevent any direct exposure of the nanoribbon to the Ga^+^ ions.

### 4.4 DW Motion Measurements

Current‐driven DW dynamics were measured using a magneto‐optical Kerr microscope. Linearly polarized light, polarized transverse to the incident direction was focused perpendicularly onto the sample. Up (⨀) and down (⨂) domains display opposite Kerr rotation angles so that the transitions between two oppositely magnetized domains, the DWs, are readily identified. Electrical contacts were bonded to the contact pads nearby the nanoribbon (Figure S1). The DW displacement was obtained by comparing the DW position before and after each series of current pulses. Unless otherwise stated, one pulse series contains ten current pulses with a pulse duration of 10 ns, with a 100 µs spacing between successive pulses. Three pulse series formed one measurement cycle. The DW velocity was calculated as:

$$v=\frac{\Delta q}{N_{\mathrm{pulse}}t_{\mathrm{pulse}}}$$

where Δ*q* is the DW displacement, *N*_pulse_ is the total number of pulses in a measurement cycle, and *t*_pulse_ is the individual pulse duration. The DW velocity is found from the mean value from at least two measurement cycles. During the MOKE measurements, the focal plane of the incident beam was adjusted to lie between the maximum and minimum surface heights to optimize the imaging quality of the 3D devices.

### 4.5 Oersted Field Calculations

The current‐generated Oersted fields were calculated from the Biot‐Savart Law:

$$\vec{B}=\frac{\mu_{0}}{4\pi }\cdot \frac{I\cdot d\vec{l}\times \vec{r}}{r^{3}}=\frac{\mu_{0}}{4\pi }\int \frac{\vec{J}\times \left(r-r^{\prime }\right)}{{\left(r-r^{\prime }\right)}^{3}}dV$$

where *I* is the current, and *r* and *r*′ indicate the position within the volume *V* of the nanoribbon. We used a current density of *J* = 1.5 × 10^8^ A cm^−2^. The field ${\vec{H}}_{\text{Oe}}$ is given by $\vec{B}/\mu_{0}$:

$${\vec{H}}_{\text{Oe}}=\frac{1}{2\pi }\int \int \frac{\vec{J}\times \left(r-r^{\prime }\right)}{{\left(r-r^{\prime }\right)}^{2}}\mathrm{dydz}=\frac{1}{2\pi }\sum_{i,j}J\Delta y\Delta z\;\frac{\hat{x}\times \left(r_{0}-r_{\mathrm{ij}}\right)}{{\left(r_{0}-r_{\mathrm{ij}}\right)}^{2}}$$

We used Δ*y* = 2 nm and Δ*z* = 0.3 nm to define the cross‐sectional area of each element with the current flowing along $\hat{x}$. Note that the nanoribbon length (along the x‐direction) is orders of magnitude larger than its width (y‐direction) and thickness (z‐direction). The cross‐section profile of the nanoribbon stays constant along the x‐direction. Thus, the system can be treated as a 2D solution of infinite length, as depicted in Figure 4. Next, we include the conductivities, σ_*i*_, of Pt, Co, and Ni, so that the current densities *J_i_* in each of these layers are given by,

$$J_{i}=\sigma_{i}\frac{\bar{J}\cdot t}{\sum_{i}\sigma_{i}\cdot t_{i}}$$

where *t* is the total thickness, and σ_*i*_, *t_i_* represent the conductivity of each metal and the corresponding layer thickness. We use conductivities given in www.periodictable.com. For the calculations for the various nanoribbons, we used the geometrical shape corresponding to the actual device.

### 4.6 CIDWM Simulations

A modified 1D DW motion model (*q*‐ϕ‐χ model) was carried out with the parameters listed in Table S1. The detailed description of the model is given in Supporting Information Note 1.

### 4.7 Statistical Analysis

The DW velocity statistics were based on multiple (*n*) measurements in the same device. The final DW velocity is the average over at least two repeated measurement cycles. The statistical error bars indicate the standard error calculated through: $\sigma_{\mathrm{DW}}=\sqrt{\frac{\sum_{c}{\left(v_{c}-\bar{v}\right)}^{2}}{n}}$. The DMI field was extracted from linear fits of the measured DW velocity versus magnetic field applied along x, carried out for different DW configurations (⨀⨂ or ⨂⨀) and different current polarities (+*J* or −*J*). The reported DMI fields correspond to the average value obtained from measurements for +*J* and −*J*, and the uncertainty was estimated from the standard error of the two extracted intercepts corresponding to +*J* and −*J*.

## Author Contributions

S.S.P.P. initiated and supervised the project. T.D., Y.G., S.‐H.Y., and S.S.P.P. contributed to the design of the experiments. T.D. made the 3D devices using the system developed by A.M.A.F., and J.D. deposited the thin films. T.D. performed the SEM characterizations and FIB preparations. T.D. and Y.G. performed the MOKE measurements. C.F. and T.D. carried out the Oersted field simulations. S.‐H.Y. developed the analytical model of the DW motion. T.D., S.‐H.Y., and S.S.P.P. wrote the manuscript. All authors discussed and commented on the manuscript.

## Conflicts of Interest

The authors declare no conflicts of interest.

## Supporting information




**Supporting File**: adma74919‐sup‐0001‐SuppMat.docx.

## Data Availability

The data that support the findings of this study are available from the corresponding author upon reasonable request.
