# Supplementary material for: Current‐Induced Domain Wall Dynamics in Pt/Co/Ni/Co Nanoribbons With Curvilinear Cross‐Sections
Source: Adv Mater. 2026 Sep 24;38(62):e74919. doi: 10.1002/adma.74919 (PMC13741136; doi:10.1002/adma.74919)
Supplement: Supplementary file 1 [file ADMA-38-e74919-s001.docx]

Supporting Information

Current-induced domain wall dynamics in Pt/Co/Ni/Co nanoribbons with curvilinear cross-sections

Tiange Dong, Jitul Deka, Chi Fang, André M. A. Farinha, Yicheng Guan, See-Hun Yang, and Stuart S. P. Parkin^*^

This file includes:

Supplementary Figures S1-S23

Supplementary Table S1

Supplementary Note 1-3

The following abbreviations are used:

CV:	convex

CC:	concave

DW:	domain wall

PMA:	perpendicular magnetic anisotropy

SEM:	scanning electron microscope

FIB:	focused ion beam

SHE:	spin Hall effect

DMI:	Dzyaloshinskii–Moriya interaction

IP:	in-plane

OOP:	out-of-plane

**Figure S1. Scanning electron microscope characterization of 3D curvilinear nanoribbons.** Images of (**a**) CV/CC and (**b**) CC/CV nanoribbons. The CV and CC segments are colored in blue and green, respectively. Scale bars: 5 μm. **c** Schematic diagrams of CV/CC (left) and CC/CV (right) devices showing the direction of +*J*. **d** and (**e**) show SEM images of the bonding of the electrical wires to extended regions connected to the two ends of the nanoribbon that are electrically isolated by FIB cuts, as indicated in (d). The red-shaded region electrically connects to the positive electrode and the blue-shaded region electrically connects to the negative electrode. Scale bars: 50 μm. **f** shows (left column) SEM images of flat, concave, and convex nanoribbons (top to bottom) and (right column) CV/CC and CC/CV nanoribbons (top to bottom). Scale bars: 5 μm.

**Figure S2. Illustration of advanced multiphoton lithography (MPL) system.** **a** A 780 nm femtosecond laser and a 532 nm continuous wave (CW) laser are transmitted through the glass substrate from its backside to initiate polymerization of the photoresist placed on the frontside of the glass substrate. **b** Layer-by-layer printing with a small between-layer spacing (left), and a large between-layer spacing (right). The former can fabricate high-precision nanoribbons with well-defined curvatures. The light orange region represents a convex cross-sectional design, and the light blue transparent ellipsoid represents the polymerized voxels.

**Figure S3. Current-induced DW motion on nanoribbons with opposite curvatures.** A series of MOKE images from (a) flat, (b) convex, and (c) concave nanoribbons. A sequence of eight current pulses with *J*=1.42×10^8^ A cm⁻² and pulse lengths of 10 ns are applied between each MOKE image. Black and white contrast show the displacement of ⨀⨂ and ⨂⨀ DWs, respectively. Note that (**a**) shows the motion of two DWs, a ⨀⨂ and a ⨂⨀ DW (from left to right) in series, which are introduced into the nanoribbon. The same DW velocity and the same tilting angle are measured. The ⨀⨂ and ⨂⨀ DWs move at the same velocity with similar tilting angles but in opposite directions. Scale bars correspond to 5 μm.

**Figure S4. Simulations of the current-induced Oersted field.** (**a)** and (**b**) show the magnetic field lines and the absolute value of the Oersted field distributed across the nanoribbon cross-section for *J* = 1.5×10^8^ A cm. (**c)** to (**g**) show the field strength (color code shown on right hand side) and magnetic field lines (arrows) for flat, convex, concave, CV/CC, and CC/CV nanoribbons, respectively (top to bottom). The black line shows the cross-section of each nanoribbon. The Oersted field is directed to the right (+y direction) mostly at and above the top surface, and is directed to the left (-y direction) mostly at and below the bottom surface. For the positions near the left and right edges, the Oersted field contains a component along z.

**Figure S5. Calculated Oersted field along y,** $\boldsymbol{H}_{\mathbf{Oe}}^{\mathbf{y}}$**, at the top surface of the nanoribbon for various *J*,** $\boldsymbol{\kappa}$**, and *w*.** $H_{\mathrm{Oe}}^{y}$ are calculated for several values of *J* and $\kappa$ that are shown in (**a**) and (**c**) for the convex nanoribbons and (**b**) and (**d**) for the concave nanoribbons. All $H_{\mathrm{Oe}}^{y}$ are positive, corresponding to the +y direction in **Figure 4**, and **Figure S4**. The calculated $H_{\mathrm{Oe}}^{y}$ for nanoribbons of varying *w* are shown in (**e**). The cross-sectional curvature is fixed at $\kappa=\pm$ 0.1 μm^-1^ for (**a**), (**b**), and (**e**). The current is fixed at *J* = 1.5×10^8^ A cm⁻² for (**c**), (**d**), and *J* = 2×10^8^ A cm⁻² for (**e**).

**Figure S6. Calculated Oersted field along z,** $\boldsymbol{H}_{\mathbf{Oe}}^{\mathbf{z}}$**.** $H_{\mathrm{Oe}}^{z}$ are calculated and shown for (**a**) flat, convex, concave nanoribbons (left to right), and, (**b**) CV/CC and CC/CV nanoribbons (left to right). Positive $H_{\mathrm{Oe}}^{z}$ (red) corresponds to the field direction pointing up, and negative $H_{\mathrm{Oe}}^{z}$ (blue) corresponds to the field direction pointing down. The magnitude of $H_{\mathrm{Oe}}^{z}$ is very small except near the left and right edges of the nanoribbon.

**Figure S7. Illustration of the geometrical effect on the DW velocity at** $-$***J*. a** and (**b**) illustrate the DW motion in convex nanoribbons (blue), while (**c**) and (**d**) illustrate the DW motion in concave nanoribbons (green). A ⨀⨂ DW is shown in (**a**) and (**c**), and a ⨂⨀ DW is shown in (**b**) and (**d**), which are the same as the ⨀⨂ DW and ⨂⨀ DW for +*J* in **Figure 5**. **e** and (**f**) illustrate the DW motion in CV/CC nanoribbons, and (**g**) and (**h**) illustrate the DW motion in CC/CV nanoribbons, respectively. For $-$*J* the Oersted field and the SHE injection are opposite to the +*J* case.

**Figure S8. Calculation of the DW velocity *v* and tilting** $\boldsymbol{\chi}$ **at** $-$***J*.** The velocity and tilt angle of a ⨀⨂ DW are shown in (**a**) and (**c**), respectively and for a ⨂⨀ DW in (**b**) and (**d**), respectively. As for the +*J* case, both ⨀⨂ and ⨂⨀ DWs move faster and tilt less on the concave nanoribbons than on the convex nanoribbons. $\chi$ is reversed in sign for $-$*J* as compared with +*J*.

**Figure S9. Magnetic hysteresis loops measured from Pt/Co/Ni/Co thin films deposited on a glass substrate.** **a** Out-of-plane magnetization and (**b**) in-plane magnetization versus magnetic field, from which the saturation magnetization, *M*_S_, is deduced to be ~450 emu cm^-3^, and the perpendicular anisotropy, *H*_k0_*_,_*to be ~10^4^ Oe. The magnetic coercivity, *H*_C_, is ~75 Oe, which is approximately half that measured on the flat 3D devices. This is attributed to the lower surface roughness and large area of the glass substrate as compared to that of the 3D device.

**Figure S10. SEM images of racetrack nanoribbons with reduced *w*.** SEM images of nanoribbons with *w* = 2 μm (upper) and 800 nm (lower) are shown. The V-shaped bars on the right side are used for electrical isolation. Scale bars: 5 μm.

**Figure S11. DW nucleation by OOP magnetic field**. Panels (**i**)-(**iv**) show the nucleation of a single ⨀⨂ DW and its subsequent current-induced motion, whereas panels (**v**)-(**viii**) show the same procedure for a single ⨂⨀ DW. A large OOP field is first applied to magnetize the entire sample in (**i**) and (**v**). A smaller reversed OOP field is then used to nucleate a single DW at the left end of the wire in (**ii**) and (**vi**). The external field is removed and current pulses are applied to drive the DW in (**iii**), (**iv**), and (**vii**), (**viii**). Background subtraction is performed before each image. Scale bars: 5 μm.

**Figure S12. DW nucleation by IP magnetic field and current pulses**. Panels (**i**)-(**iii**) show the nucleation of a single ⨀⨂ DW and its current-induced motion, whereas panels (**iv**)-(**vi**) show the same procedure for a ⨂⨀ DW. The sample is first magnetized into a single-domain state. An IP field is applied either to the left or right, and a series of current pulses is applied simultaneously as shown in (**i**) and (**iv**). Because the DMI fields of ⨀⨂ and ⨂⨀ DWs are opposite, the IP field can break the symmetry and selectively nucleate one DW configuration. The IP field is then removed, and current pulses are applied to drive the DW, as shown in (**ii**), (**iii**), and (**v**), (**vi**). Scale bars: 5 μm.

**Figure S13. CIDWM in single-curvature nanoribbons.** DW velocities for nanoribbons with $\kappa$ $=\pm$0.1 μm^-1^ measured under (**a**) +*J* and (**b**) $-$*J*. Red and blue symbols indicate the velocities of ⨀⨂ and ⨂⨀ DWs, respectively. The velocity ratio $\frac{v_{\mathrm{cc}}}{v_{\mathrm{cv}}}$ in (**c**) is extracted from (**a**) and (**b**) and is ~1.1~1.3.

**Figure S14. DW velocities under externally applied field** $\boldsymbol{H}_{\mathbf{y}}^{\boldsymbol{0}}$. (**a)** and (**b**) velocities for ⨀⨂ and ⨂⨀ DWs, shown in red and blue, respectively. $H_{y}^{0}$ is oriented across the nanoribbon. Positive values correspond to a left-to-right direction in the cross-sectional view (i.e., +y direction in Figure S4), the same direction as the current-generated Oersted field at the nanoribbon top surface. The color indicates the magnitude of $H_{y}^{0}$.

**Figure S15. Exemplary images of current induced DW displacement for various** $\boldsymbol{H}_{\mathbf{y}}^{\boldsymbol{0}}$. Black contrast indicates the displacement of a ⨀⨂ DW in (**i**)-(**iii**), and white contrast indicates the displacement of a ⨂⨀ DW in (**iv**)-(**vi**) for a sequence of 10 current pulses, each 10 ns long. Dashed lines are a guide to the eye and show the position of the corresponding DW under the same sequence of current pulses in zero external field. *J* =1.47 ×10^8^ A·cm^-^². Scale bars: 5 μm.

**Figure S16. Cross-sectional SEM characterization of the deposited HM/FM thin films on 3D nanoribbons**. Cross-sectional images of (**a**)-(**c**) a flat ribbon and (**d**)-(**f**) a concave ribbon. A Pt protective layer is first deposited on top of the nanoribbon before FIB. The dark area is the 3D polymer scaffold, while the bright intermediate layer corresponds to the HM/FM thin film. The middle and right panels show a magnified view of the dashed square region in the left panel. Scale bars from left to right are: 2μm, 1μm, and 500 nm. The film thickness is generally uniform across the width. On the other hand, the absolute cross-sectional profile of the device is strongly affected by FIB preparation and electron- and/or ion-beam exposure ^1^.

**Figure S17.** **2θ-θ XRD scan measured on a HM/FM multilayered thin film deposited on a glass substrate that is also used for fabrication of the nanoribbons.**

**Note 1. CIDWM simulations**

We simulate the following DW parameters: DW position $q$, DW magnetization angle $\phi$, and DW tilt angle $\chi$ for ⨀⨂ or ⨂⨀ DW configurations using a developed 1D DW model. Different cross-sectional curvatures are introduced by their corresponding Oersted field, $\vec{H}_{\mathrm{Oe}}$. Although $\vec{H}_{\mathrm{Oe}}$ is spatially non-uniform in the cartesian coordinate system, it is oriented approximately tangential to the curved surface of the device, as shown in the $\vec{H}_{\mathrm{Oe}}$ profile simulations in Figure 4 and S4. Consequently, $\vec{H}_{\mathrm{Oe}}$ can be considered oriented along the y-direction at each point in the curvilinear local coordinate system. This enables us to simplify the full spatially varying Oersted-field profile by an effective uniform transverse field $\vec{H}_{Oe}^{y}$ in our simulations. The differential equations corresponding to the DW motion can be written as ^1-4^:

$\dot{q}=\frac{1}{a_{d}\cos\chi}\left[ -\frac{\alpha\gamma d_{n}}{2M_{S}}P\left( q \right)-\frac{\gamma d_{n}}{2}H_{k}\sin\left( 2\left( \phi-\chi\right) \right)-\frac{\gamma\pi}{2}\alpha d_{n}H_{\mathrm{SHE}}\cos\phi+\frac{\gamma\pi}{2}d_{n}H_{\mathrm{Oe}}^{y}\sin\left( \phi-\phi_{y} \right)+\frac{\gamma\pi}{2}d_{n}H_{\mathrm{DMI}}\sin\left( \phi-\chi-\phi_{\mathrm{DMI}} \right) \right]$

$\dot{\phi}=\frac{1}{a_{d}}\left[ -\frac{\gamma}{2M_{s}}P\left( q \right)+\frac{\gamma\alpha}{2}H_{k}\sin\left( 2\left( \phi-\chi\right) \right)-\frac{\alpha\gamma\pi}{2}H_{\mathrm{Oe}}^{y}\sin\left( \phi-\phi_{y} \right)-\frac{\gamma\pi}{2}H_{\mathrm{SHE}}\cos\phi-\frac{\alpha\gamma\pi}{2}H_{\mathrm{DMI}}\sin\left( \phi-\chi-\phi_{\mathrm{DMI}} \right) \right]$

$\dot{\chi}=\frac{1}{C(\chi)}\left[ -\left( \frac{4H_{k0}}{M_{S}}+H_{k}\cos^{2} \left( \phi-\chi\right)-\pi H_{\mathrm{Oe}}^{y}\cos\left( \phi-\phi_{y} \right)-\pi H_{\mathrm{DMI}}\cos\left( \phi-\chi-\phi_{\mathrm{DMI}} \right) \right)\tan\chi+\pi H_{\mathrm{DMI}}\sin\left( \phi-\chi-\phi_{\mathrm{DMI}} \right)-H_{k}\sin\left( 2\left( \phi-\chi\right) \right) \right]$

Here, $a_{d}=1+\alpha^{2}$. $\phi_{y}$ and $\phi_{\mathrm{DMI}}$ are the angle of the Oersted field and the DMI field, respectively. All other parameters are explained and taken from **Table S1**. $d_{n}$ defines the effective DW width, $P\left( q \right)$ corresponds to a perpendicular effective field, and $C(\chi)$ is a coefficient. These are given by:

$d_{n}=\frac{\Delta}{\sqrt{1+\frac{H_{k}{c\mathrm{os}}^{2}\left( \phi-\chi\right)-H_{\mathrm{Oe}}^{y}\sin\phi}{H_{k0}}}}$

$P\left( q \right)=-2H_{z}M_{s}+2V\sin(\frac{2\pi q}{q_{0}})$

$$C\left( \chi\right)=\frac{\pi^{2}}{6\gamma}\alpha\left[ {(\frac{w\sec\chi}{d_{n}\pi})}^{2}+\tan^{2}\chi\right]$$

The out-of-plane external field $H_{z}$ is zero in our case. And a pinning potential for the DW is introduced as a sinusoidal function with amplitude $V$ and period $q_{0}$, which corresponds with the threshold current for CIDWM.

The current *J* can be converted to a SHE effective field $H_{\mathrm{SHE}}= \frac{\hbar\theta_{\mathrm{SH}}J}{2eM_{S}t_{\mathrm{FM}}}$ with a spin Hall angle $\theta_{\mathrm{SH}}$ set to 0.1. The DW width is given by $\Delta=\sqrt{A/K_{\mathrm{eff}}}$ whereas $K_{\mathrm{eff}}=\frac{1}{2}\mu_{0}M_{S}H_{k0}$. Values of $M_{S}$ and $H_{k0}$ are extracted from **Figure S9**.

**Table S1. Parameters for DW velocities and tilting angle calculation.**

| **Parameter** | **Value** |
| --- | --- |
| Gamma, $\gamma$ | 1.761×10^7^ s^-1^Oe^-1^ |
| Damping factor, $\alpha$ | 0.1 |
| Pulse duration, *T*_pulse_ | 240 ns (24 pulses ×10 ns) |
| Wire width, *w* | 4 μm |
| DW width, $\Delta$ | 5 nm |
| FM layer thickness, *t*_FM_ | 1.3 nm |
| Exchange stiffness, *A* | 10 pJm^-1^ |
| Saturation magnetization, *M*_S_ | 450 emucm^-3^ |
| Perpendicular anisotropy, *H*_k0_ | 10^4^ Oe |
| Shape anisotropy, *H*_k_ | 50 Oe |
| DMI effective field, *H*_DMI_ | 474 Oe |
| Spin hall angle, $\theta_{\mathrm{SH}}$ | 0.1 |
| Spin Hall field, $H_{\mathrm{SHE}}$ | 0-900 Oe |
| Pinning potential amplitude, *V* | 3.7×10^5^ ergcm^-3^ |
| Pinning potential period, *q*0 | 50 nm |

**Note 2. Thin film strain estimate**

To estimate the strain in the multilayered thin film, we first define and calculate the position of the neutral plane, which corresponds to zero strain. For the TaN/Pt/Co/Ni (120/50/3/7/3 Å) multilayer stack, the neutral plane lies approximately at the center of the stack at ~*t*/2, due to the similar biaxial strain $B_{i}=\frac{E_{i}}{1-v_{i}}$ for Co/Ni, Pt, and TaN ^5,6^. Here $E_{i}$ is the Young’s modulus and $v_{i}$ is the Poisson ratio of the material. For a nanoribbon with lateral width $w$=4 μm, the effective lateral width $w_{i}$ at a layer position $z_{i}$ (measured along the surface normal direction) can be written as:

$$w_{i}=\frac{w}{R}\left( R+z_{i} \right)$$

where $R$ is the radius of curvature of the nanoribbon. Note that the multilayers are ultrathin compared with the radius of curvature of the nanoribbon. The corresponding strain $\varepsilon_{i}$ along the lateral direction is then given by:

$$\varepsilon_{\mathrm{yy},i}=\frac{w_{i}-w_{\mathrm{neu}}}{w_{\mathrm{neu}}}=\frac{z_{i}-z_{\mathrm{neu}}}{R+z_{\mathrm{neu}}}$$

where $z_{\mathrm{neu}}$ is the position of the neutral plane. The sign of the strain is opposite for CV and CC geometries. Therefore, the maximum estimated strains at the top Co layer are $+$9.15$\times$10^-4^ and $-$9.16$\times$10^-4^ for the CV and CC structures, respectively. Since the FM layers are ultrathin and located very close to the neutral plane, corresponding to a small $z_{i}-z_{\mathrm{neu}}$, the resulting curvature-induced strain is very small ^6,7^. In addition, the estimated strain at the Pt/Co interface is $\pm$3$\times$10^-5^. Considering the lattice constants of Pt (2.77 Å), Co (2.51 Å), and Ni (2.49 Å) ^8^, this strain is negligible compared with the intrinsic lattice mismatch at the Pt/Co interfaces. Therefore, the geometry-induced strain is not expected to produce any observable modification of the PMA or interfacial DMI in our devices ^9,10^. Consistent with this estimate, the measured hysteresis loops and DMI fields are nearly identical for the flat, CV and CC devices, as shown in Figure 1c and **Figure 2g**.

**Note 3. DW energy calculation**

We now calculate the energy and curvature-related energy modifications of a DW in nanoribbons with curved cross-sectional geometries. The total DW energy $E_{\mathrm{DW}}$ is written as:

$${{E_{\mathrm{DW}}=E_{\mathrm{ex}}+E}_{\mathrm{PMA}}+E}_{\mathrm{Dip}}+E_{\mathrm{DMI}}+E_{\mathrm{Oe}}$$

which includes exchange, PMA, dipolar, DMI, and Oersted-field contributions. To describe the CV and CC curved geometries, we introduce local curvilinear coordinates ($\boldsymbol{e}_{x}$, $\boldsymbol{e}_{s}$, $\boldsymbol{e}_{n}$), as illustrated in **Figure S18**. Therefore:

$$\boldsymbol{e}_{x}=\hat{x}$$

$$\boldsymbol{e}_{s}=\frac{\partial\boldsymbol{r}}{\partial s}=\cos\left( \kappa s \right)\hat{y}+\sin\left( \kappa s \right)\hat{z}$$

$$\boldsymbol{e}_{n}=\boldsymbol{e}_{x}\times\boldsymbol{e}_{s}=-\sin\left( \kappa s \right)\hat{y}+\cos\left( \kappa s \right)\hat{z}$$

where ($\hat{x}$, $\hat{y}$, $\hat{z}$) are the cartesian unit vectors, $s$ is the arc-length coordinate along the curved cross-sectional surface, and $\kappa$ is the cross-sectional curvature. We define $\kappa$ > 0 for CV nanoribbons and $\kappa$ < 0 for CC nanoribbons. Since the curvature is only in the cross-sectional plane, the local basis does not vary along the x-direction, so that $\frac{\partial\boldsymbol{e}_{s}}{\partial x}$ = 0 and $\frac{\partial\boldsymbol{e}_{n}}{\partial x}$ = 0.

**Figure S18. Schematic of the nanoribbon geometry. (a)** Flat, (**b**) CV, and (**c**) CC nanoribbon geometries are shown, respectively. (**d)** and (**e**) are the local curvilinear coordinate systems ($\boldsymbol{e}_{x}$, $\boldsymbol{e}_{s}$, $\boldsymbol{e}_{n}$) for CV and CC nanoribbons shown in a cross-sectional view, respectively.

The magnetization $\boldsymbol{m}$ inside the DW is given by:

$$\boldsymbol{m}=\sin\theta\cos\phi\boldsymbol{e}_{x}+\sin\theta\sin\phi\boldsymbol{e}_{s}+\cos\theta\boldsymbol{e}_{n}$$

where $\theta$is the polar angle and $\phi$ is the azimuthal angle. The DW tilts by an angle $\chi$ ^11,12^. $\theta$ is defined with respect to $\hat{X}$, which is the DW normal vector:

$$\theta\left( X \right)=2\tan^{-1} \exp\left( p\frac{X}{\Delta} \right)$$

$$X=\left( x-q \right)\cos\chi+s\sin\chi$$

here $q$ is the DW center position, $X$ is the distance from the calculation point to the DW center, $\Delta$ is the DW width parameter, and $p$ is the DW polarity. For ⨀⨂ DWs $p$ = $+$1 and for ⨂⨀ DWs $p$ = $-$1. The DW effective lateral length $w_{\mathrm{DW}}=w\sec\chi.$

We next evaluate each energy contribution to the DW energy and identify the corresponding curvature-induced effect. The perpendicular magnetic anisotropy easy axis is assumed to follow the local surface normal ^7^. The PMA energy per unit film thickness is therefore given by:

$$E_{\text{PMA}}=K_{\text{eff}}\int\sin^{2} \theta dx ds=w\sec\chi\Delta2K_{\text{eff}}$$

$$K_{\text{eff}}=K_{\mathrm{PMA}}-\frac{1}{2}\mu_{0}M_{s}^{2}$$

under the assumption that the PMA contribution is not directly modified by $\kappa$ which is consistent with the experimentally measured hysteresis loop (Figure 1c).

In the local curvilinear basis ($\boldsymbol{e}_{x}$, $\boldsymbol{e}_{s}$, $\boldsymbol{e}_{n}$), the basis vectors vary along $\hat{s}$ according to $\partial_{s}\boldsymbol{e}_{s}={\kappa\boldsymbol{e}}_{n}$ and $\partial_{s}\boldsymbol{e}_{n}={-\kappa\boldsymbol{e}}_{s}$ that includes the sign of the curvature. For the DMI energy:

$$\nabla\cdot\boldsymbol{m}=\partial_{x}m_{x}+\partial_{s}m_{s}-\kappa m_{n}$$

$$\sigma_{\text{DMI}}=-D\left[ m_{n}\nabla\cdot\boldsymbol{m}-\left( \boldsymbol{m}\cdot\nabla\right)m_{n} \right]$$

$$=-D[\partial_{x}\theta\cos\phi+\partial_{s}\theta\sin\phi-\kappa\cos^{2} \theta]$$

where $D$ is the material-defined DMI constant. The total DMI energy of a DW is given by:

$$E_{\text{DMI}}=-D\cos\left( \phi-\chi\right)\int_{-\infty}^{+\infty} \frac{d\theta}{dX}dX-D\kappa\int_{-\infty}^{+\infty} \sin^{2} \theta dX=w\sec\chi[-p\pi D\cos\left( \phi-\chi\right)-2D\kappa\Delta]$$

where the first term is the conventional DMI energy of a flat nanoribbon, and the second term is a curvature-related energy correction that is linear in $\kappa$. Interestingly, this term arises from the geometrical rotation of the local curvilinear basis and is independent of the DW configuration and magnetization (i.e., $\frac{\partial2D\kappa\Delta}{\partial p}$ and $\frac{\partial2D\kappa\Delta}{\partial\phi}=0$). Compared with the flat case, this curvature-related contribution is much weaker by a factor $\kappa\Delta$, since $\left| \kappa\Delta\right|\ll$1 by a curvature of μm^-1^ and $\Delta$ of a few nanometers for our devices. This explains why the measured DMI field remains nearly unchanged among the flat, CV, and CC devices (Figure 2g).

The exchange energy density is first written in the standard form:

$$\sigma_{\text{ex}}=A_{\mathrm{ex}}\left| \nabla\boldsymbol{m} \right|^{2}$$

with $A_{\mathrm{ex}}$ the exchange stiffness. For a DW that is approximately uniform along its lateral length, the expansion of $\left| \nabla\boldsymbol{m} \right|^{2}$ and the corresponding DW exchange energy is:

$$\left| \nabla\boldsymbol{m} \right|^{2}=\left( \partial_{x}\theta\right)^{2}+\left( \partial_{s}\theta\right)^{2}-2\kappa\left( \partial_{s}\theta\right)\sin\phi+\kappa^{2}\left( \cos^{2} \theta+\sin^{2} \theta\sin^{2} \phi\right)$$

$$E_{\text{ex}}=w\sec\chi\left[ \frac{2A_{ex}}{\Delta}+2\pi pA_{ex}\kappa\sin\chi\sin\phi+2A_{\mathrm{ex}}\kappa^{2}\Delta\cos^{2} \phi\right]$$

Two $\kappa$-induced exchange terms appear in this equation. The linear term (~$\kappa$) has a DMI-like character, that depends on $p$, $\chi$, and $\phi$, and will favor one of the particular DW chiralities. The quadratic term (~$\kappa^{2}$) has an anisotropy-like character in $\cos^{2} \phi$ (uniaxial symmetry) and is symmetric for $+\kappa$ and $-\kappa$. Note that the linear term and the quadratic term favor different magnetization orientations. The former favors $\phi$ = 90° or 270°, while the latter favors $\phi$ = 0° or 180°. In the present PMA system, the interfacial DMI is the dominant factor that controls the DW chirality, making $\phi$ = 0° or 180° and $\chi$ relatively small. Thus, the DMI-like exchange is also small relative to the anisotropy-like term, unless $\left| \kappa\right|$ is substantially large to reconfigure the DW magnetization and configurations.

For dipolar interactions, a rigorous calculation of the dipolar energy requires contributions from surface and volume charges ^1^:

$$E_{\text{Dip}}=\frac{\mu_{0}}{8\pi}\iint\frac{\sigma_{m}\left( \boldsymbol{r} \right)\sigma_{m}\left( \boldsymbol{r}\boldsymbol{'} \right)}{\left| \boldsymbol{r}-\boldsymbol{r}^{'} \right|} dSdS'+\frac{\mu_{0}}{8\pi}\iint\frac{\rho_{m}\left( \boldsymbol{r} \right)\rho_{m}\left( \boldsymbol{r}\boldsymbol{'} \right)}{\left| \boldsymbol{r}-\boldsymbol{r}\boldsymbol{'} \right|} dVdV'+\frac{\mu_{0}}{4\pi}\iint\frac{\rho_{m}\left( \boldsymbol{r} \right)\sigma_{m}\left( \boldsymbol{r}\boldsymbol{'} \right)}{\left| \boldsymbol{r}-\boldsymbol{r}\boldsymbol{'} \right|} dVdS'$$

The surface-surface charge mainly affects the $K_{\text{eff}}$ that has been included in the PMA energy, while the volume-volume charge gives rise to the DW shape anisotropy. In the curved geometry, the volume magnetic charge density is:

$$\rho_{m}=-M_{s}\frac{d\sin\theta}{dX}\cos(\phi-\chi)+M_{s}\kappa m_{n}$$

And its corresponding dipolar energies are:

$$E_{\text{Dip,k}}=w\sec\chi2K_{\text{DW}}\Delta\cos^{2} \left( \psi-\chi\right)$$

$$E_{Dip, cur} =w\sec\chi K_{\text{cur}}p\kappa\Delta\cos(\phi-\chi)+\delta(\kappa^{2})$$

where $K_{\text{DW}}$ is the DW shape anisotropy coefficient and $K_{\text{cur}}$ is the effective curvature-induced coefficient. Therefore, $E_{\text{Dip, k}}$ describes the conventional DW shape anisotropy in the flat nanoribbon, whereas $E_{Dip, cur}$ is the curvature-induced correction. In $E_{Dip, cur}$, the linear term in $\kappa$ is constrained by symmetry that reversing the curvature sign and the DW polarity maps a ⨀⨂ DW in a CV nanoribbon onto a ⨂⨀ DW in a CC nanoribbon, and vice versa. Previous studies indicate that curvature-induced dipolar effects become pronounced only for *R* below 100 nm ^13-15^, corresponding to around 10 μm^-1^. In our devices of $\kappa$~0.1 μm^-1^, the contribution of the curvature-induced dipole energy should also be very small.

Finally, we consider the contribution from the spatially distributed Oersted field. In the curvilinear basis, the relevant field component along $\boldsymbol{e}_{s}$ with the energy of:

$$\sigma_{\mathrm{Oe}}=-\mu_{0}M_{s}H_{\mathrm{Oe}}^{s}\left( s \right)\sin\theta\cos(\phi-\phi_{\mathrm{Oe}})$$

$$E_{\mathrm{Oe}}=-\mu_{0}M_{s}\int H_{\mathrm{Oe}}^{s}\left( s \right)\sin\theta\cos(\phi-\phi_{\mathrm{Oe}}) dx ds=-\mu_{0}M_{s}\pi\Delta\sec\chi\cos(\phi-\phi_{\mathrm{Oe}}) \int_{-w/2}^{w/2} H_{\mathrm{Oe}}^{s}\left( s \right) ds$$

if the Oersted field is expressed in the Cartesian y-directional field $H_{\mathrm{Oe}}^{y}\left( s \right)$, it becomes:

$$E_{\mathrm{Oe}}=-\mu_{0}M_{s}\pi\Delta\sec\chi\cos(\phi-\phi_{\mathrm{Oe}}) \int_{-w/2}^{w/2} H_{\mathrm{Oe}}^{y}\left( s \right)\cos(\kappa s) ds$$

$H_{\mathrm{Oe}}^{y}\left( s \right)$ corresponds to the lateral Oersted field profile in the Cartesian coordinates calculated and shown in Figure 4. Its spatial distribution depends on the cross-sectional geometry (i.e., approximately half-sinusoidal for single-curvature CC or CV ribbons and becomes full-sinusoidal for hybrid CC/CV or CV/CC ribbons).

We now calculate the DW energy $E_{\mathrm{DW}}$($\phi, \chi, \kappa, p, \Delta$) as a function of all DW parameters. The DW width is determined by the combined energy contributions:

$$\Delta=\sqrt{\frac{A_{ex}}{K_{\text{eff}}+K_{\text{DW}}\cos^{2} \left( \phi-\chi\right)-D\kappa{+K}_{\text{cur}}p\kappa\cos(\phi-\chi)-\frac{\pi}{2}M_{s}H_{Oe, eff}^{y}\cos(\phi-\phi_{\mathrm{Oe}})}}$$

Using the parameters listed in Table S1, we calculate the DW energy landscape by sweeping over $\phi, \chi, \kappa, p$. The results are shown in **Figure S19-S23**. We first discuss the case without considering the $H_{\mathrm{Oe}}^{y}\left( s \right)$. At a small $\kappa$ that corresponds to our experimental device geometry, all curvature-induced interactions (~$\kappa$ or ~$\kappa^{2}$) produce unnoticeable changes in either the DW width and energy or the equilibrium values of $\phi$ and $\chi$. This is reflected by the nearly unchanged energy minimal position in the ($\chi$,$\phi$) space (Figure S19 and S21). We then include the $H_{\mathrm{Oe}}^{y}\left( s \right)$ contribution. As shown in Figure S20 and S21, the equilibrium $\phi$ and $\chi$ exhibit a clearer change. When $\kappa$ changes from $-$0.2 to $+$0.2 μm^-1^, $\chi$ increases from 0.2° to 0.8° for ⨀⨂ DWs and decreases from $-$0.2° to $-$0.8° for the ⨂⨀ DWs, respectively. This trend is consistent with our experimental result that both ⨀⨂ and ⨂⨀ DW are more tilted in the CV devices than in the CC devices. Meanwhile, calculated DW energy and width also show that, within the experimental curvature range, the lateral Oersted field gives the dominant contribution compared with other curvature-induced interactions (Figure S21).

To further evaluate the role of intrinsic curvature-induced interactions, we also calculate the DW energy over a larger $\kappa$ range (0 ~ $\pm$50 μm^-1^) while excluding the lateral Oersted field. As a result, curvature-induced exchange and dipolar terms become increasingly important at a large $\left| \kappa\right|$ value (e.g., exceeds 10 μm^-1^). There, the energy difference of CV and CC devices becomes clearly distinguishable as shown in Figure S23. Interesting, for CV devices of large $+\kappa$, the ⨀⨂ and ⨂⨀ DWs tend to preserve their original left-handed Néel chirality (i.e., $\phi-\chi=$ 180° for ⨀⨂ and $\phi-\chi=$ 0° for ⨂⨀). By contrast, for CC devices of large $-\kappa$ , both ⨀⨂ and ⨂⨀ DWs are significantly deviate from the ideal $\phi-\chi=$ 180° or 0°. This arises from the curvature-induced dipolar interaction acting as a DMI-like term and favors the right-handed Néel-type chirality opposite to the interfacial DMI in CC devices ^14^.

In conclusion, our energy calculations show that, for the curvatures used in our devices, curvature-induced modifications of exchange, DMI, or dipolar energy are too small to dominate the changes in DW energy density or velocities. Instead, the curvature-modified lateral Oersted field provides the leading geometry-dependent contribution to the DW energy, and therefore, is the most plausible origin for the observed DW velocity differences in our curvilinear nanoribbons.

**Figure S19. Calculated** $\boldsymbol{E}_{\mathbf{DW}}$**(**$\boldsymbol{\phi, \chi, \kappa, p}$**) at small** $\boldsymbol{\kappa}$ **without considering the current-induced lateral Oersted field.** Black crosses indicate the energy minima in ($\chi,\phi$) space, corresponding to the most favorable $\chi$, $\phi$, $\Delta$, and DW energy for a given geometry.

**Figure S20. Calculated** $\boldsymbol{E}_{\mathbf{DW}}$**(**$\boldsymbol{\phi, \chi, \kappa, p}$**) at small** $\boldsymbol{\kappa}$ **taking into account the current-induced lateral Oersted field.** Clear changes in the position of the energy minima are shown as compared with **Figure S19**.

**Figure S21. Calculated DW** $\boldsymbol{\phi}$**,** $\boldsymbol{\chi}$**,** $\Delta$**, and DW energy as a function of** $\boldsymbol{\kappa}$**.** **a**, **b** show $\phi$ and $\chi$ for the ⨀⨂ DW, and (**c**), (**d**) show $\phi$ and $\chi$ for the ⨂⨀ DW. Black and red indicate the results excluding or including the influence of the lateral Oersted field, respectively. The latter case shows an orders of magnitude larger influence on the DW equilibrium state at a given $\kappa$ scale. **e** and **f** show the corresponding DW width and DW energy which are the same for ⨀⨂ and ⨂⨀ DWs.

**Figure S22. Calculated** $\boldsymbol{E}_{\mathbf{DW}}$**(**$\boldsymbol{\phi, \chi, \kappa, p}$**) at intermediate** $\boldsymbol{\kappa}$ **without considering** $\boldsymbol{H}_{\mathbf{Oe}}^{\mathbf{y}}$**.**

**Figure S23. Calculated** $\boldsymbol{E}_{\mathbf{DW}}$**(**$\boldsymbol{\phi, \chi, \kappa, p}$**) at large** $\boldsymbol{\kappa}$ **without considering** $\boldsymbol{H}_{\mathbf{Oe}}^{\mathbf{y}}$**.**

**References for the supplementary information**

1	Farinha, A. M. A., Yang, S.-H., Yoon, J., Pal, B. & Parkin, S. S. P. Interplay of geometrical and spin chiralities in 3D twisted magnetic ribbons. *Nature* **639**, 67-72 (2025).

2	Ryu, K.-S., Thomas, L., Yang, S.-H. & Parkin, S. Chiral spin torque at magnetic domain walls. *Nat. Nanotechnol.* **8**, 527-533 (2013).

3	Garg, C., Yang, S.-H., Phung, T., Pushp, A. & Parkin, S. S. P. Dramatic influence of curvature of nanowire on chiral domain wall velocity. *Sci. Adv.* **3**, e1602804 (2017).

4	Garg, C. *et al.* Highly Asymmetric Chiral Domain-Wall Velocities in Y-Shaped Junctions. *Nano Letters* **18**, 1826-1830 (2018).

5	Xu, H., Yang, H., Xie, Y. & Li, R.-W. Strain-Mediated Control of Magnetic Properties in Thin Films. *Nano Letters* **26**, 5585-5597 (2026).

6	Fedorov, P. *et al.* Self-assembly of Co/Pt stripes with current-induced domain wall motion towards 3D racetrack devices. *Nat. Commun.* **15**, 2048 (2024).

7	Kim, J. & Dho, J. Bending strain–induced changes in perpendicular magnetic properties of Pt/Co/Pt films on flexible substrates. *Appl. Phys. Lett.* **124** (2024).

8	Guan, Y. *et al.* Increased Efficiency of Current-Induced Motion of Chiral Domain Walls by Interface Engineering. *Adv. Mater.* **33**, 2007991 (2021).

9	Deger, C. Strain-enhanced Dzyaloshinskii–Moriya interaction at Co/Pt interfaces. *Sci. Rep.* **10**, 12314 (2020).

10	Gu, K. *et al.* Three-dimensional racetrack memory devices designed from freestanding magnetic heterostructures. *Nat. Nanotechnol.* **17**, 1065-1071 (2022).

11	Boulle, O. *et al.* Domain Wall Tilting in the Presence of the Dzyaloshinskii-Moriya Interaction in Out-of-Plane Magnetized Magnetic Nanotracks. *Phys. Rev. Lett.* **111**, 217203 (2013).

12	Yang, S.-H., Ryu, K.-S. & Parkin, S. Domain-wall velocities of up to 750 m s−1 driven by exchange-coupling torque in synthetic antiferromagnets. *Nat. Nanotechnol.* **10**, 221-226 (2015).

13	Landeros, P. *et al.* Reversal modes in magnetic nanotubes. *Appl. Phys. Lett.* **90** (2007).

14	Umetsu, N. *et al.* Spin-orbit torque driven motion of chiral domain walls induced by radial magnetization in nanotube geometry. *Phys. Rev. B* **112**, 094425 (2025).

15	Sheka, D. D. A perspective on curvilinear magnetism. *Appl. Phys. Lett.* **118** (2021).
